# Comparison of clinical characteristics and outcomes of patients with coronavirus disease 2019 at different ages

**DOI:** 10.18632/aging.103298

**Published:** 2020-06-04

**Authors:** Mengmeng Zhao, Menglong Wang, Jishou Zhang, Jian Gu, Pingan Zhang, Yao Xu, Jing Ye, Zhen Wang, Di Ye, Wei Pan, Bo Shen, Hua He, Mingxiao Liu, Menglin Liu, Zhen Luo, Dan Li, Jianfang Liu, Jun Wan

**Affiliations:** 1 Department of Cardiology, Renmin Hospital of Wuhan University, Wuhan, China; 2 Cardiovascular Research Institute, Wuhan University, Wuhan, China; 3 Hubei Key Laboratory of Cardiology, Wuhan, China; 4 Department of Clinical Laboratory, Renmin Hospital of Wuhan University, Wuhan, China; 5 Department of Medical Affairs, Renmin Hospital of Wuhan University, Wuhan, China; 6 Medical Quality Management Office, Renmin Hospital of Wuhan University, Wuhan, China; 7 Department of Emergency, Renmin Hospital of Wuhan University, Wuhan, China; 8 Department of Pediatrics, Renmin Hospital of Wuhan University, Wuhan, China

**Keywords:** 2019 novel coronavirus, coronavirus disease 2019, COVID-19, age, clinical characteristics, prognosis

## Abstract

Background: Information about the clinical characteristics and mortality of patients with coronavirus disease 2019 at different ages is limited.

Results: The older group had more patients with dyspnea and fewer patients with fever and muscle pain. Older patients had more underlying diseases, secondary infection, myocardial injury, renal dysfunction, coagulation dysfunction, and immune dysfunction on admission. More older patients received immunoglobulin therapy and mechanical ventilation. The proportions of patients with multiple organ injuries, critically ill patients and death increased significantly with age. The older groups had higher cumulative death risk than the younger group. Hypertension, cerebrovascular disease, comorbidities, acute cardiac injury, shock and complications are independent predictors of death.

Conclusions: The symptoms of the elderly patients were more atypical, with more comorbidities, secondary infection, organ injuries, immune dysfunction and a higher risk of critical illness. Older age was an important risk factor for mortality.

Methods: 1000 patients diagnosed with coronavirus disease 2019 from January 1, 2020 to February 14, 2020 were enrolled. According to age, patients were divided into group 1 (<60 years old), group 2 (60-74 years old) and group 3 (≥75 years old). The clinical symptoms, first laboratory results, CT findings, organ injuries, disease severity and mortality were analyzed.

## INTRODUCTION

Since the outbreak of coronavirus disease 2019 (COVID-19) in Wuhan, it has spread globally [1]. According to data from the World Health Organization (WHO), as of April 22, 2020, at least 2,471,136 patients have been confirmed to have COVID-19, of whom 169,006 have died [2]. At present, the number of patients with severe acute respiratory syndrome coronavirus 2 (SARS-CoV-2) infection is still increasing. The numbers of infected patients and deaths both exceeded the respective figures associated with the outbreaks of severe acute respiratory syndrome (SARS) in 2003 [3] and Middle East respiratory syndrome (MERS) in 2015 [4]. Compared to the mortality of SARS (10%) and MERS (35%), COVID-19 has a lower fatality rate of 2.3% [5–7]. However, the rapidly increasing number of cases and increasing evidence of human-to-human transmission suggest that SARS-CoV-2 is more contagious than SARS-CoV and MERS-CoV [8, 9].

According to a report from the Chinese Centers for Disease Control and Prevention, older patients over 60 years old in Wuhan accounted for 44.1% of COVID-19 patients [9]. A recent study [10] from the Lancet indicated that the proportion of patients over 60 years old in Wuhan was significantly higher than that in the rest of China, which reflected a more severe illness in Wuhan. Older patients had a substantially higher case fatality ratio of 6.4% than younger patients. Increasing evidence has shown that elderly patients are more likely to develop severe illness and have higher risks of in-hospital death [11–14]. However, there were no reports about the clinical findings of patients diagnosed with SARS-CoV-2 infection at different ages. The mechanisms of the poor prognosis in elderly patients remain unclear.

In this study, we analyzed and compared the clinical characteristics and outcomes of COVID-19 patients at different ages.

## RESULTS

### Comparison of basic clinical characteristics among the three groups

A total of 1000 COVID-19 patients were analyzed during the study period, including 473 young patients (47.3%) (age group 1), 359 elderly patients (35.9%) (age group 2) and 168 super-aged patients (16.8%) (age group 3). A total of 466 (46.6%) were male, and 534 (53.4%) were female. The median (interquartile ranges) interval from disease onset to admission of the three groups was 10 (7-14), 11 (8-15) and 10 (6-13) days, respectively. The most common symptoms before admission of all the patients were fever (75.4%), followed by cough (59.7%), fatigue (33.5%) and dyspnea (25.5%). The percentage of patients with fever in age group 3 was lower than that in age group 2 (79.1% vs. 69.6%, P <0.05). With increasing age, the incidence of dyspnea increased (22% vs. 28.1% vs. 29.8%), and the rate of muscle pain decreased (3.6% vs. 5% vs. 8.9%). No significant difference was found for the other symptoms, such as cough, chest pain, catarrhal symptoms, fatigue, dizziness, headache and digestive symptoms (Table 1). The finger oxygen saturation on admission increased with age (median, 96% vs. 97% vs. 98%). During hospitalization, 623 patients (66.3%) had fever, and the proportion of patients with fever (59.7% vs. 71.9% vs. 73.2%) and shortness of breath (respiratory rate > 30 times per minute) (9.5% vs. 17.5% vs. 19.6%) increased with age (Table 2).

**Table 1:** Baseline characteristics of COVID-19 patients.

	**all (n=1000)**	**age<60(n=473)**	**60-74(n=359)**	**age≥75(n=168)**
**Age, median (IQR), y**	61 (46,70)	46 (34,53)	67 (64,70)*	80 (77,84)*#
**Male (n, %)**	466 (46.6%)	216 (45.7%)	164 (45.7%)	86 (51.2%)
**Female (n, %)**	534 (53.4%)	257 (54.3%)	195 (54.3%)	82 (48.8%)
**Onset of symptom to admission, median (IQR), d**	10 (7,14)	10 (7,14)	11 (8,15)*	10 (6,13)#
**Initial symptoms, No. (%)**				
**Fever**	754 (75.4%)	353 (74.6%)	284 (79.1%)	117 (69.6%)#
**Symptoms of respiratory system**
Sore throat	43 (4.3%)	31 (6.6%)	7 (1.9%)*	5 (3%)
Cough	597 (59.7%)	290 (61.3%)	202 (56.3%)	105 (62.5%)
Expectoration	190 (19%)	81 (17.1%)	65 (18.1%)	44 (26.2%)*#
Chest tightness	209 (20.9%)	78 (16.5%)	97 (27%)*	34 (20.2%)
Chest pain	19 (1.9%)	9 (1.9%)	7 (1.9%)	3 (1.8%)
Dyspnea	255 (25.5%)	104 (22%)	101 (28.1%)*	50 (29.8%)*
**Catarrhal symptoms**	20 (2%)	12 (2.5%)	5 (1.4%)	3 (1.8%)
**Neuromuscular symptoms**	388 (38.8%)	199 (42.1%)	127 (35.4%)	62 (36.9%)
Fatigue	335 (33.5%)	172 (36.4%)	111 (30.9%)	52 (31%)
Dizziness	33 (3.3%)	15 (3.2%)	10 (2.8%)	8 (4.8%)
Headache	32 (3.2%)	19 (4%)	7 (1.9%)	6 (3.6%)
Lethargy	10 (1%)	6 (1.3%)	0 (0%)	4 (2.4%)#
Muscle ache	66 (6.6%)	42 (8.9%)	18 (5%)*	6 (3.6%)*
**Digestive symptoms**				
Anorexia	134 (13.4%)	61 (12.9%)	49 (13.6%)	24 (14.3%)
Nausea	21 (2.1%)	8 (1.7%)	8 (2.2%)	5 (3%)
Vomiting	26 (2.6%)	11 (2.3%)	8 (2.2%)	7 (4.2%)
Abdominal pain	7 (0.7%)	3 (0.6%)	3 (0.8%)	1 (0.6%)
Diarrhea	100 (10%)	48 (10.1%)	35 (9.7%)	17 (10.1%)

The total number of patients with available data: n(<60)=473, n(60-74)=359, n(≥75)=168. * p<0.05 vs. <60 group. # p<0.05 vs. 60-74 group.

**Table 2:** Vital signs and comorbidity of COVID-19 patients.

	**all (n=1000)**	**age<60(n=473)**	**60-74(n=359)**	**age≥75(n=168)**
**Characteristics on admission**				
Fever, No. (%)^***a***^	200 (21.3%)	98 (22.1%)	71 (20.8%)	31 (20.3%)
Temperature	36.7 (36.4,37.1)	36.7 (36.4,37.1)	36.7 (36.5,37.1)	36.7 (36.5,37)
Temperature, No. (%)^***a***^
< 37.3°C	739 (78.7%)	346 (77.9%)	271 (79.2%)	122 (79.7%)
37.3-38.0°C	117 (12.5%)	55 (12.4%)	46 (13.5%)	16 (10.5%)
38.1-39.0°C	72 (7.7%)	35 (7.9%)	23 (6.7%)	14 (9.2%)
≥ 39.1°C	11 (1.2%)	8 (1.8%)	2 (0.6%)	1 (0.7%)
Heart rate, median (IQR), bpm ^***b***^	82 (76,92)	82(76,92)	83(76,92)	80(76,89)
Systolic pressure, median (IQR), mmHg ^***c***^	126 (116,139)	123(112,132)	130(120,142)*	131(117,148)*
Diastolic pressure, median (IQR), mmHg ^***d***^	76 (69,83)	75(68,80)	76(70,84)*	76(67,84)
Respiratory rate, median (IQR), bpm ^***e***^	20 (18,20)	19(18,20)	20(18,21)*	19(18,21)
Finger oxygen saturation, median (IQR), % ^***f***^	97 (95,99)	98(96,99)	97(95,99)*	96(92,98)*#
**Characteristics during hospital admission, No. (%)**				
Fever	623 (66.3%)	265 (59.7%)	246 (71.9%)*	112 (73.2%)*
Highest temperature ^***g***^				
< 37.3°C	316 (33.7%)	179 (40.3%)	96 (28.1%)*	41 (26.8%)*
37.3-38.0°C	352 (37.5%)	150 (33.8%)	145 (42.4%)*	57 (37.3%)
38.1-39.0°C	203 (21.6%)	76 (17.1%)	85 (24.9%)*	42 (27.5%)*
≥ 39.1°C	67 (7.1%)	38 (8.6%)	16 (4.7%)*	13 (8.5%)
>41.0°C	1 (0.1%)	1 (0.2%)	0 (0%)	0 (0%)
Respiratory rate≥30 bpm	141 (14.1%)	45 (9.5%)	63 (17.5%)*	33 (19.6%)*
**Comorbidity, No. (%)**				
Diabetes	118 (11.8%)	34 (7.2%)	55 (15.3%)*	29 (17.3%)*
Hypertension	282 (28.2%)	62 (13.1%)	131 (36.5%)*	89 (53%)*#
Coronary heart disease	60 (6%)	6 (1.3%)	29 (8.1%)*	25 (14.9%)*#
COPD	23 (2.3%)	0 (0%)	17 (4.7%)*	6 (3.6%)*
Asthma	12 (1.2%)	3 (0.6%)	5 (1.4%)	4 (2.4%)
Cerebrovascular disease	32 (3.2%)	3 (0.6%)	8 (2.2%)*	21 (12.5%)*#
Chronic renal disease	24 (2.4%)	7 (1.5%)	7 (1.9%)	10 (6%)*#
Chronic liver disease	29 (2.9%)	12 (2.5%)	12 (3.3%)	5 (3%)
Malignancy	28 (2.8%)	7 (1.5%)	11 (3.1%)	10 (6%)*
Autoimmune disease	13 (1.3%)	3 (0.6%)	6 (1.7%)	4 (2.4%)
Organ transplantation	2 (0.2%)	2 (0.4%)	0 (0%)	0 (0%)
Only one comorbidity	237 (23.7%)	67 (14.2%)	115 (32%)*	55 (32.7%)*
≥2 comorbidities	168 (16.8%)	34 (7.2%)	75 (20.9%)*	59 (35.1%)*#
With comorbidity	405 (40.5%)	101 (21.4%)	190 (52.9%)*	114 (67.8%)*#

The total number of patients with available data: a: n(<60)=444, n(60-74)=342, n(≥75)=153; b: n(<60)=443, n(60-74)=352, n(≥75)=153; c: n(<60)=351, n(60-74)=314, n(≥75)=143; d: n(<60)=354, n(60-74)=314, n(≥75)=143; e: n(<60)=443, n(60-74)=342, n(≥75)=152; f: n(<60)=319, n(60-74)=302, n(≥75)=136; g: n(<60)=444, n(60-74)=342, n(≥75)=153. * p<0.05 vs. <60 group. # p<0.05 vs. 60-74 group.

In addition, 405 patients (40.5%) had at least one comorbidity. The most common comorbidity was hypertension (28.2%), followed by diabetes (11.8%), coronary artery disease (CAD) (6%) and cerebrovascular disease (CVD) (3.2%). The proportion of patients with any comorbidity, more than one comorbidity, diabetes, hypertension, CAD, CVD and chronic renal disease all increased with advancement of age (Table 2).

### Results of laboratory tests and CT scans

We collected and analyzed the initial laboratory tests after admission. According to the routine blood test results, the median white blood cell counts and neutrophil counts increased with age, while the lymphocyte counts decreased with age. The proportion of patients with lymphopenia in the older groups was higher than that in the younger group (43.1% vs. 62% vs. 70.7%). The probability of thrombocytopenia increased with age (5.3% vs. 11.3% vs. 21%) (Table 3).

**Table 3:** General laboratory findings of COVID-19 patients on admission to hospital.

	**Median (IQR)**			
	**all (n=1000)**	**age<60(n=473)**	**60-74(n=359)**	**age≥75(n=168)**
**Blood routine ^a^**				
White blood cell count, × 10^9^/L	5.48 (4.12,7.23)	5.16 (3.97,6.65)	5.72 (4.26,7.47)*	5.89 (4.53,8.45)*
<3.5, No. (%)	136 (13.9%)	77 (16.8%)	39 (11%)*	20 (12%)
>9.5, No. (%)	106 (10.8%)	37 (8.1%)	37 (10.4%)	32 (19.2%)*#
Neutrophil count, × 10^9^/L	3.67 (2.48,5.44)	3.16 (2.22,4.6)	4.06 (2.74,5.97)*	4.51 (3.11,7.29)*#
Neutrophil %	68.9 (57.9,81.85)	64.6 (53.6,75.4)	72 (62.7,82.75)*	80 (66,86.8)*#
Lymphocyte count, × 10^9^/L	1.02 (0.72,1.46)	1.2 (0.88,1.6)	0.93 (0.65,1.39)*	0.81 (0.53,1.13)*#
<1.1, No. (%)	535 (54.6%)	197 (43.1%)	220 (62%)*	118 (70.7%)*
Lymphocyte %	20.6 (11.6,30.15)	24.9 (16.9,34.7)	18.5 (10.55,26.35)*	13 (7.5,22)*#
Platelet count, × 10^9^/L	208 (160,267.5)	209 (166,264)	215 (163.5,283.5)*	186 (137,233)*
<125, No. (%)	99 (10.1%)	24 (5.3%)	40 (11.3%)*	35 (21%)*#
>350, No. (%)	78 (8%)	32 (7%)	42 (11.8%)*	4 (2.4%)*#
Red blood cell count, × 10^10^/L	4.07 (3.71,4.45)	4.28 (3.94,4.65)	3.99 (3.64,4.25)*	3.81 (3.38,4.22)*#
**Liver function ^b^**				
Alanine aminotransferase, U/L	24 (16,42)	25 (16,47)	24 (17,39)	23 (15,38)
>150, No. (%)	17 (1.7%)	10 (2.2%)	5 (1.4%)	2 (1.2%)
Aspartate aminotransferase, U/L	28 (20,40)	25 (20,37)	29 (21,40)*	31.5 (22,46)*#
>120, No. (%)	15 (1.5%)	7 (1.5%)	4 (1.1%)	4 (2.4%)
Total bilirubin, μmol/L ^**c**^	10.5 (7.9,14.13)	10.1 (7.48,13.4)	10.45 (8.03,14.1)	11.95 (8.65,16.27)*#
Direct bilirubin, μmol/L	3.8 (2.7,5.1)	3.5 (2.5,4.8)	3.9 (2.8,5.1)*	4.6 (3.4,6.6)*#
Total protein, g/L	60.9 (57.3,64.63)	62.2 (58.48,65.9)	59.65 (56.43,63.78)*	59.85 (55.7,63.08)*
Albumin, g/L	36.8 (33.4,39.83)	38.65 (35.8,41.4)	35.2 (32.6,37.98)*	34.4 (31.9,37.2)*#
Globulin, g/L	23.73 (21.6,26.8)	23.2 (21.3,25.7)	24.15 (21.7,27.2)*	24.85 (22.03,27.7)*
**Kidney function ^d^**				
Creatinine, μmol/L	60 (49,73)	56 (47.75,70.25)	60 (50,70)*	69.5 (54.25,98.75)*#
Increase, No. (%)	116 (11.9%)	29 (6.4%)	34 (9.6%)	53 (31.9%)*#
Blood urea nitrogen, nmol/L	4.6 (3.59,6.2)	4.06 (3.15,5.04)	4.91 (3.81,6.5)*	6.95 (4.81,11.4)*#
Uric acid, μmol/L	249 (199,327)	252.5 (201,322.25)	233 (194,300.5)*	282.5 (217.25,393.25)*#
Increase, No. (%)	168 (17.2%)	72 (15.8%)	42 (11.8%)	54 (32.5%)*#
Estimated glomerular filtration rate, mL/min	98.86 (88.36,111.87)	112.98 (102.88,121.29)	94.87 (88.55,99.75)*	79.85 (53.68,88.94)*#
≤90, No. (%)	273 (27.9%)	43 (9.4%)	100 (28.2%)*	130 (78.3%)*#
**Injury of cardiac and skeletal muscle**				
Creatine kinase, U/L ^e^	61 (38,104.25)	58 (37,101)	60.5 (38,100)	67 (42,138.25)*#
>310, No. (%)	60 (6.3%)	25 (5.7%)	15 (4.2%)	20 (12.2%)*#
Creatine kinase-myocardial band isoenzyme, ng/mL ^***f***^	0.97 (0.64,1.79)	0.68 (0.48,0.97)	1.11 (0.81,1.88)*	1.85 (1.17,3.24)*#
>5, No. (%)	35 (4.6%)	4 (2%)	11 (3.5%)	20 (13.3%)*#
Lactate dehydrogenase, U/L ^***g***^	264 (203.75,361.25)	236 (187,312)	284 (221.25,368.75)*	298 (222,439)*
>250, No. (%)	523 (54.5%)	197 (44.7%)	217 (61.3%)*	109 (66.1%)*
Myoglobin, μg/L ^h^	44.54 (28.5,85.05)	33.27 (21.95,54.42)	44 (30.76,75.99)*	88.56 (55.55,207.42)*#
>110, No. (%)	132 (17.5%)	28 (9.6%)	43 (13.8%)	61 (40.7%)*#
Hypersensitive troponin I, ng/mL ^i^	0.006 (0.006,0.018)	0.006 (0.006,0.006)	0.006 (0.006,0.017)*	0.028 (0.01,0.077)*#
>0.0796, No. (%)	66 (8.7%)	7 (2.4%)	22 (7.1%)*	37 (24.3%)*#
Cholinesterase, U/L ^j^	8127 (6469.5,9827)	9091 (7521.75,10688.5)	7666.5 (6124.25,9072.25)*	6060 (4775.5,7030.5)*#
>11900	49 (8.2%)	39 (13.1%)	9 (4.5%)*	1 (1%)*
**Arterial Blood Gas Analysis ^k^**				
Blood PH	7.42 (7.38,7.45)	7.42 (7.38,7.45)	7.43 (7.38,7.46)	7.42 (7.37,7.46)
<7.35, No. (%)	77 (13.6%)	23 (11%)	35 (15.2%)	19 (15.1%)
>7.45, No. (%)	176 (31.1%)	56 (26.8%)	81 (35.1%)	39 (31%)
Arterial oxygen saturation, %	96 (91,98)	97 (92,98)	96 (92,98)	95 (89,98)*
<95%, No. (%)	222 (39.2%)	64 (30.6%)	96 (41.6%)*	62 (49.2%)*
Arterial partial pressure of oxygen, mmHg	80 (60,105)	87 (62,107)	78 (62,102)	73 (55.25,99)*
<60 mmHg, No. (%)	140 (24.7%)	49 (23.4%)	52 (22.5%)	39 (31%)
Arterial partial pressure of carbon dioxide, mmHg l	40 (36,45)	42 (38,45)	40 (35.5,44)*	37 (34,42)*#
<35 mmHg, No. (%)	111 (20.1%)	23 (11.3%)	49 (21.6%)*	39 (32%)*#
>45 mmHg, No. (%)	153 (27.7%)	71 (35%)	55 (24.2%)*	27 (22.1%)*
Lactic acid, mmol/L m	2.1 (1.6,2.8)	2.1 (1.6,2.8)	2.1 (1.6,2.8)	2.2 (1.5,2.88)
**Electrolytes ^n^**				
K+, mmol/L	3.84 (3.43,4.25)	3.88 (3.54,4.3)	3.8 (3.38,4.2)*	3.81 (3.4,4.21)
Na+, mmol/L	139.2 (136.1,142)	139.6 (137,142)	139 (136,142)	139 (135,142)
Cl -, mmol/L ^o^	105.6 (102.8,107.8)	105.7 (103.28,107.5)	105.4 (102.7,107.8)	105.2 (102.35,108.38)
**Coagulation function ^p^**				
Prothrombin time activity, % q	84 (74.9,93.3)	84.7 (76,96.75)	84.7 (76,92.8)*	78.85 (71.3,89.7)*#
Activated partial thromboplastin time, s	28.4 (26.2,31.2)	28.5 (26.35,31)	27.9 (25.6,31)*	29.05 (27.1,32)*#
D-dimer, μmol/L	0.81 (0.41,2.35)	0.51 (0.29,1.41)	0.93 (0.48,2.41)*	1.77 (0.7,5.49)*#

The total number of patients with available data: a: n(<60)=457, n(60-74)=355, n(≥75)=167; b: n(<60)=457, n(60-74)=355, n(≥75)=166; c: n(<60)=456, n(60-74)=354, n(≥75)=166; d: n(<60)=456, n(60-74)=355, n(≥75)=166; e: n(<60)=442, n(60-74)=354, n(≥75)=164; f: n(<60)=200, n(60-74)=312, n(≥75)=150; g: n(<60)=442, n(60-74)=354, n(≥75)=165; h: n(<60)=291, n(60-74)=312, n(≥75)=150; i: n(<60)=297, n(60-74)=312, n(≥75)=152; j: n(<60)=298, n(60-74)=202, n(≥75)=99; k: n(<60)=209, n(60-74)=231, n(≥75)=126; l: n(<60)=203, n(60-74)=227, n(≥75)=122; m: n(<60)=209, n(60-74)=230, n(≥75)=126; n: n(<60)=458, n(60-74)=357, n(≥75)=167; o: n(<60)=456, n(60-74)=355, n(≥75)=166; p:n(<60)=351, n(60-74)=337, n(≥75)=160; q: n(<60)=352, n(60-74)=338, n(≥75)=160. * p<0.05 vs. <60 group. # p<0.05 vs. 60-74 group.

Aspartate aminotransferase, direct bilirubin, globulin creatinine, blood urea nitrogen, uric acid, creatinine kinases MB isoenzyme (CKMB), hypersensitive troponin I (hs-TnI) and lactate dehydrogenase (LDH) all increased with advancement of age. The proportion of patients with increased aspartate aminotransferase (>120 U/L), CKMB (>5 ng/mL), hs-TnI (>0.0796 ng/mL), LDH (>250 U/L) and decreased estimated glomerular filtration (≤90 mL/min) all increased with age. The older group had more patients with myocardial injury, renal dysfunction, and liver dysfunction on admission (Table 3).

The results of arterial blood gas analysis showed that older patients presented with lower arterial oxygen saturation and arterial partial pressure of oxygen. No difference was discovered in blood pH among the three age groups. The coagulation function results showed that the activated partial thromboplastin time and D-dimer (median, 0.51 vs. 0.93 vs. 1.77 μmol/L) increased with age, but prothrombin time activity decreased with age (Table 3).

Regarding the biomarkers of nonspecific inflammation, the results showed that the levels of biomarkers including C-reactive protein (CRP) (median, 11.4 vs. 40 vs. 54.3 mg/L), high-sensitivity C-reactive protein (hs-CRP) (median, 5 vs. 5 vs. 5 mg/L) and procalcitonin (PCT) (median, 0.05 vs. 0.06 vs. 0.12 ng/mL) increased with age. The proportion of patients with elevated CRP, elevated serum amyloid protein (SAA), and elevated PCT all significantly increased with age, suggesting that the percentage of elderly patients with secondary infection was significantly increased. Interleukin 6 (IL-6) expression increased (median, 5.71 vs. 7.19 vs. 16.66 pg/mL) with age, although there were no significant differences in interferon-γ, IL-2, IL-5, IL-10 or tumor necrosis factor among the three groups. Older patients had higher levels of complement C3, and C4 and immunoglobulin A, G, and M than younger patients. In addition, the counts of CD19 cells, CD3 cells, CD4 cells (median, 412 vs. 328 vs. 266/μL) and CD8 cells (median, 263 vs. 177 vs. 120.5/μL) decreased with age (Table 4).

**Table 4:** Inflammatory response and immunoreaction of COVID-19 patients on admission to hospital.

	**Median (IQR)**			
	**all (n=1000)**	**age<60(n=473)**	**60-74(n=359)**	**age≥75(n=168)**
**Nonspecific inflammation index**				
C-reactive protein, mg/L ^***a***^	29.1 (5,70.9)	11.4 (5,46.98)	40 (7.35,83.85)*	54.3 (18.55,87.6)*#
>10, No. (%)	615 (64.8%)	226 (51.6%)	250 (72%)*	139 (84.8%)*#
High-sensitivity C-reactive protein, mg/L ^***b***^	5 (5,5)	5 (1.75,5)	5 (5,5)*	5 (5,5)*#
>5, N0. (%)	138 (14.7%)	64 (14.8%)	46 (13.3%)	28 (17.2%)
Serum amyloid protein, mg/L ^c^	59.42 (5,202.77)	9.94 (5,127.66)	200 (66.43,300)*	200 (61.48,300)*
>10, No. (%)	148 (61.2%)	78 (49.1%)	44 (83%)*	26 (86.7%)#
Erythrocyte sedimentation rate, mm/h ^***d***^	54 (31,71)	40.5 (28,62.5)	62 (41,86)*	57.5 (39.75,83.5)*
Procalcitonin, ng/mL ^***e***^	0.06 (0.04,0.13)	0.05 (0.03,0.1)	0.06 (0.04,0.13)*	0.12 (0.06,0.3)*#
>0.5, No. (%)	62 (7.4%)	13 (3.8%)	23 (7%)	26 (16.3%)*#
**Cytokines ^*f*^**				
Interferon-γ, pg/mL	3.82 (2.72,5.53)	3.78 (3.01,6.02)	4 (2.81,5.4)	3.76 (2.16,4.58)
interleukin 2, pg/mL	3.49 (3.08,4.06)	3.48 (3.08,4.06)	3.55 (3.08,4.06)	3.2 (2.87,3.84)
interleukin 4, pg/mL	3.61 (3.01,4.16)	3.75 (3.16,4.29)	3.55 (3.01,4.1)	3.17 (2.81,3.85)*
Interleukin 5, pg/mL ^***g***^	2.25 (2.13,2.35)	2.23 (2.13,2.34)	2.22 (2.11,2.42)	2.31 (2.31,2.33)
Interleukin 6, pg/mL ^***h***^	7.04 (2.89,18.15)	5.71 (2.11,10.74)	7.19 (2.61,18.02)	16.66 (7.03,39.16)*#
interleukin 10, pg/mL	5.72 (4.75,7.38)	5.72 (4.74,7)	5.66 (4.59,7.4)	6.25 (4.93,7.54)
Tumor necrosis factor, pg/mL	3.11 (2.73,4.32)	3.06 (2.64,4.03)	3.26 (2.91,4.26)	3.04 (2.75,4.57)
**Humoral immunity ^*i*^**				
Complement 3, g/L	1 (0.86,1.15)	1.02 (0.86,1.17)	1.01 (0.88,1.14)	0.95 (0.81,1.06)*#
Complement 4, g/L	0.25 (0.19,0.33)	0.26 (0.19,0.34)	0.24 (0.18,0.32)*	0.25 (0.2,0.32)
Immunoglobulin A, g/L	2.34 (1.76,3.01)	2.17 (1.64,2.61)	2.44 (1.85,3.21)*	2.68 (2.03,3.4)*
Immunoglobulin E, IU/mL	43.65 (18.3,122)	45.95 (18.3,131)	42.3 (18.3,115)	42.3 (18.3,120.5)
Immunoglobulin G, g/L	11.85 (10.03,14.2)	11.5 (9.9,13.58)	12.2 (10.2,14.6)*	12.5 (10.3,14.85)*
Immunoglobulin M, g/L	0.94 (0.68,1.23)	1.01 (0.74,1.29)	0.9 (0.66,1.2)*	0.84 (0.58,1.15)*
**Cellular immunity ^j^**				
CD16+56, %	13.24 (8.52,20.58)	11.64 (7.54,18.08)	14.1 (9.26,20.29)*	16.82 (11.24,29.9)*#
CD16+56 counts, No./μL	118 (73,183)	117 (75.25,175)	118 (72.5,188)	118.5 (70,190.75)
CD19, %	15.29 (11.23,20.81)	14.92 (10.83,20.04)	16.32 (11.93,22.04)*	14.6 (10.15,21.76)#
CD19 counts, No./μL	136 (88,200)	142.5 (103,212.25)	137 (81.5,203.5)*	107 (60.25,151.75)*#
CD3, %	66.95 (57.01,74.25)	69.66 (61.85,75.67)	64.84 (56.05,72.72)*	61.24 (48.06,70.32)*#
CD3 counts No./μL	597 (378.5,904.5)	740 (508.25,1038.5)	552 (345.5,819)*	416 (243,639)*#
CD4, %	39.56 (31.64,46.06)	39.55 (32.52,45.65)	40.64 (32.76,47.79)	35.82 (29.07,45.89)*#
CD4 counts, No./μL	359 (217,548)	412 (273.5,613.75)	328 (209.5,531.5)*	266 (138,392.5)*#
CD8, %	22.17 (15.9,29.25)	25.21 (19.49,31.01)	20.19 (14.35,26.08)*	18.6 (12.12,24.99)*
CD8 counts, No./μL	211 (114,332)	263 (163.25,403.75)	177 (95,285)*	120.5 (64.75,230.5)*#
CD4/CD8	1.74 (1.24,2.65)	1.56 (1.16,2.12)	2.04 (1.4,3.07)*	1.93 (1.28,3.17)*

The total number of patients with available data: a: n(<60)=438, n(60-74)=347, n(≥75)=164; b: n(<60)=431, n(60-74)=347, n(≥75)=163; c: n(<60)=159, n(60-74)=53, n(≥75)=30; d: n(<60)=50, n(60-74)=39, n(≥75)=20; e: n(<60)=345, n(60-74)=329, n(≥75)=160; f: n(<60)=87, n(60-74)=69, n(≥75)=26; g: n(<60)=32, n(60-74)=15, n(≥75)=3; h: n(<60)=167, n(60-74)=165, n(≥75)=66; i: n(<60)=374, n(60-74)=309, n(≥75)=139; j: n(<60)=390, n(60-74)=319, n(≥75)=142. * p<0.05 vs. <60 group. # p<0.05 vs. 60-74 group.

A total of 545 CT results were collected within 3 days before or after admission. A total of 509 patients (93.4%) had pneumonia. Compared with younger patients, older patients were more likely to have bilateral involvement and paving stone/reticulation/linear findings (Table 5).

**Table 5:** Initial pulmonary CT findings of COVID-19 patients.

**Characteristics of lung CT, No. (%)**	**all (n=545)**	**age<60 (n=304)**	**60-74 (n=179)**	**age≥75 (n=62)**
Pneumonia	509 (93.4%)	279 (91.8%)	174 (97.2%)*	56 (90.3%)
Unilateral lung	73 (13.4%)	60 (19.7%)	9 (5%)*	4 (6.5%)*
Bilateral lung	454 (83.3%)	227 (74.7%)	169 (94.4%)*	58 (93.5%)*
Ground-glass opacity	405 (74.3%)	227 (74.7%)	141 (78.8%)	37 (59.7%)*#
Paving stone/reticular/linear	141 (25.9%)	62 (20.4%)	58 (32.4%)*	27 (43.5%)*
Consolidation shadow	74 (13.6%)	46 (15.1%)	25 (14%)	3 (4.8%)*
Air bronchogram	59 (10.8%)	27 (8.9%)	27 (15.1%)*	5 (8.1%)

Abbreviation: CT: computerized tomography. * p<0.05 vs. <60 group. # p<0.05 vs. 60-74 group.

### Complications after admission and treatment

Of the 1000 patients, 191 patients (19.1%) experienced complications. The most common complication was acute cardiac injury (11.6%), followed by shock (8.1%) and acute liver injury (6.4%). The rates of patients with any complication, more than one complication, acute cardiac injury, shock and liver injury were higher in the older group than in the younger group. The proportion of patients with acute cardiac injury increased with age (4% vs. 12.5% vs. 31%). Shock and acute liver injury were most likely to occur in age group 3 (Table 6).

**Table 6:** Complications and treatments of COVID-19 patients.

	**all (n=1000)**	**age<60(n=473)**	**60-74(n=359)**	**age≥75(n=168)**
**Complications, No. (%)**				
Shock	81 (8.1%)	26 (5.5%)	29 (8.1%)	26 (15.5%)*#
Acute cardiac injury	116 (11.6%)	19 (4%)	45 (12.5%)*	52 (31%)*#
Acute renal injury	29 (2.9%)	6 (1.3%)	12 (3.3%)*	11 (6.5%)*
Acute liver injury	64 (6.4%)	36 (7.6%)	23 (6.4%)	5 (3%)*
≥1 complication	191 (19.1%)	62 (13.1%)	68 (18.9%)*	61 (36.3%)*#
Only one complication	125 (12.5%)	49 (10.4%)	40 (11.1%)	36 (21.4%)*#
≥2 complications	66 (6.6%)	13 (2.7%)	28 (7.8%)*	25 (14.9%)*#
**Admission to ICU, No. (%)**	63 (6.3%)	20 (4.2%)	26 (7.2%)	17 (10.1%)*
**ICU treatment duration, day,**	7 (3,11)	7 (4,11)	10 (5,12)	4 (2,7)#
Median (IQR)				
**Oxygen therapy, No. (%)**				
Nasal catheter oxygen inhalation	661 (66.1%)	270 (57.1%)	261 (72.7%)*	130 (77.4%)*
Mask oxygen inhalation	314 (31.4%)	120 (25.4%)	120 (33.4%)*	74 (44%)*#
HFBHTI	91 (9.1%)	29 (6.1%)	35 (9.7%)	27 (16.1%)*#
Non-invasive mechanical ventilation	147 (14.7%)	46 (9.7%)	57 (15.9%)*	44 (26.2%)*#
Invasive mechanical ventilation	43 (4.3%)	15 (3.2%)	18 (5%)	10 (6%)
**Medical treatment, No. (%)**				
Antiviral treatment	927 (92.7%)	435 (92%)	340 (94.7%)	152 (90.5%)
Antibiotic treatment	783 (78.3%)	362 (76.5%)	288 (80.2%)	133 (79.2%)
Antifungal treatment	32 (3.2%)	11 (2.3%)	15 (4.2%)	6 (3.6%)
Glucocorticoids	500 (50%)	220 (46.5%)	197 (54.9%)*	83 (49.4%)
Immunoglobulin therapy	513 (51.3%)	219 (46.3%)	200 (55.7%)*	94 (56%)*
**Special treatment, No. (%)**				
CRRT	15 (1.5%)	6 (1.3%)	6 (1.7%)	3 (1.8%)
ECMO	2 (0.2%)	2 (0.4%)	0 (0%)	0 (0%)
ALSS	10 (1%)	6 (1.3%)	4 (1.1%)	0 (0%)

Abbreviation: ALSS: artificial liver support system; CRRT: continuous renal replacement therapy; ECMO: extracorporeal membrane oxygenation; ICU: intensive care unit; IQR: interquartile range. P values indicate differences between male and female patients. * p<0.05 vs. <60 group. # p<0.05vs. 60-74 group.

Regarding the treatment measures, 63 (6.3%) patients were sent to the intensive care unit (ICU). Older patients were more likely to be admitted to the ICU than younger patients. Patients in age groups 2 and 3 were more likely to receive nasal catheter oxygen inhalation, mask oxygen inhalation and noninvasive mechanical ventilation than patients in age group 1. Regarding medical treatment, the major treatments were antiviral treatment (92.7%), antibiotic treatment (78.3%), glucocorticoids (50%) and immunoglobulin therapy (51.3%). The proportion of patients receiving immunoglobulin therapy increased significantly with increasing age. No difference was found in patients receiving special supporting treatments, including continuous renal replacement therapy (CRRT), extracorporeal membrane oxygenation (ECMO), or artificial liver support system (ALSS) (Table 6).

### Clinical classification and prognosis of COVID-19 patients

The proportion of patients with critical conditions increased (18% vs. 27.3% vs. 50.6%) with advancement of age. A total of 119 patients (11.9%) died, and 197 patients (19.7%) were discharged or transferred to isolation points during hospitalization. The percentage of deaths increased with age (5.1% vs. 11.7% vs. 31.5%). However, there was no significant difference in the duration from admission to death or duration from disease onset to death among the three groups (Table 7).

**Table 7:** Clinical classification and prognosis of COVID-19 patients.

	**all (n=1000)**	**age<60(n=473)**	**60-74(n=359)**	**age≥75(n=168)**
**Clinical classification, No. (%)**				
Mild-Moderate	385 (38.5%)	250 (52.9%)	105 (29.2%)*	30 (17.9%)*#
Severe	347 (34.7%)	138 (29.2%)	156 (43.5%)	53 (31.5%)*#
Critical	268 (26.8%)	85 (18%)	98 (27.3%)*	85 (50.6%)*#
**Prognosis, No. (%) or Median (IQR)**				
Death	119 (11.9%)	24 (5.1%)	42 (11.7%)*	53 (31.5%)*#
Onset of disease to death, d	17 (13,21)	17 (13.8,20)	17 (13,21)	16 (12,22)
From hospitalization to death, d	6 (3,10)	7 (4,10)	6 (3,9)	7 (3,11)
Discharge or Transfer to the isolation point	197 (19.7%)	134 (28.3%)	52 (14.5%)*	11 (6.5%)*#
Staying in hospital	673 (67.3%)	310 (65.5%)	259 (72.1%)*	104 (61.9%)#

Abbreviation: d: day; IQR: interquartile range. * p<0.05 vs. <60 group. # p<0.05vs. 60-74 group.

**Figure 1:**
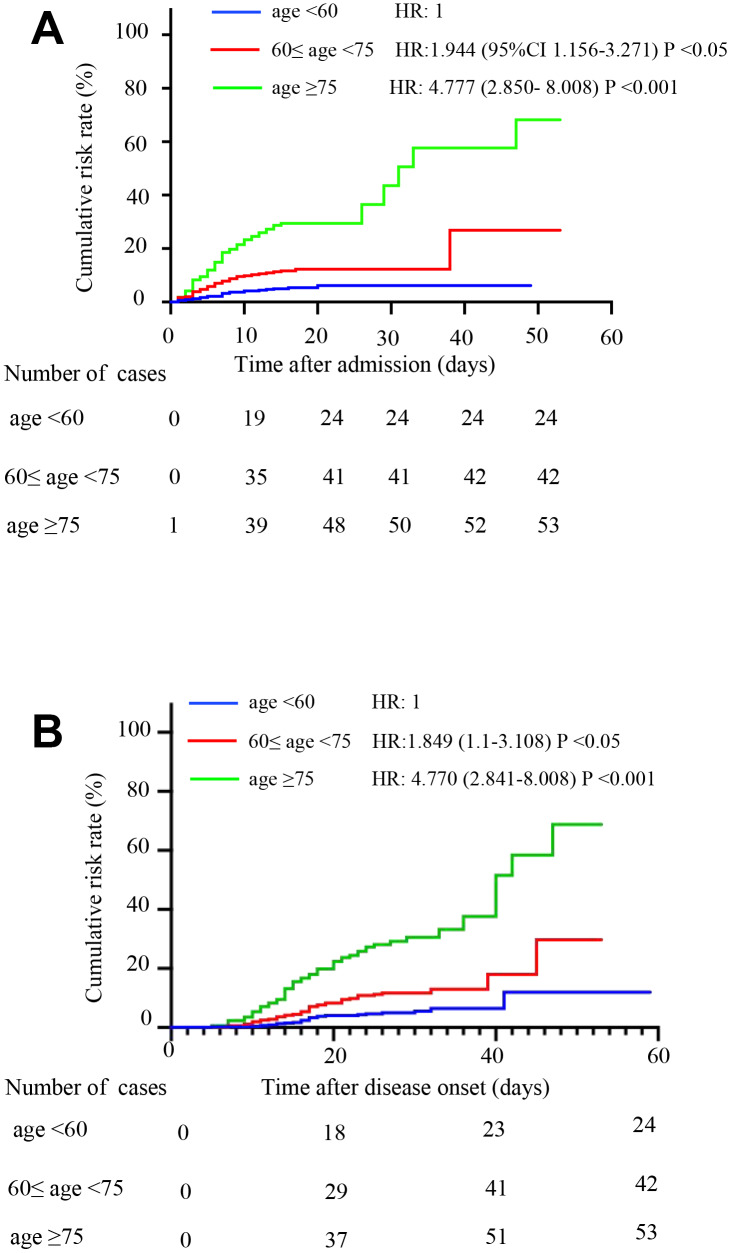
**Comparison of the time-dependent risk of death.** (**A**) The cumulative death risk after admission in age group 1 (blue curve), age group 2 (red curve) and age group 3 (green curve). Compared to age group 1, the hazard ratios (HRs) and 95% confidence intervals (95% CIs) of age groups 2 and 3 were HR: 1.944 (1.156-3.271; P <0.05) and HR: 4.777 (2.850- 8.008; P <0.001), respectively. The model was adjusted for sex and comorbidities. (**B**) The cumulative death risk after disease onset in age group 1 (blue curve), age group 2 (red curve) and age group 3 (green curve). Compared to age group 1, the HRs (95% CIs) of age groups 2 and 3 were HR: 1.849 (1.1-3.108; P <0.05) and HR: 4.77 (2.841-8.008; P <0.001). The model was adjusted for sex and comorbidities.

Age group 3 had a higher cumulative death risk than group 2 (p<0.05), which had a higher risk than group 1 (p<0.05). After adjusting for sex and comorbidity status, patients in group 2 (HR, 1.944, 95% CI, 1.156-3.271) and group 3 (HR, 4.777, 95% CI, 2.850- 8.008) were more likely to die than patients in group 1 according to observation from admission. Patients in group 2 (HR, 1.849, 95%CI 1.1-3.108) and group 3 (HR, 4.770, 95%CI, 2.841-8.008) were more likely to die according to observation from disease onset (Figure 1A and 1B).

**Figure 2:**
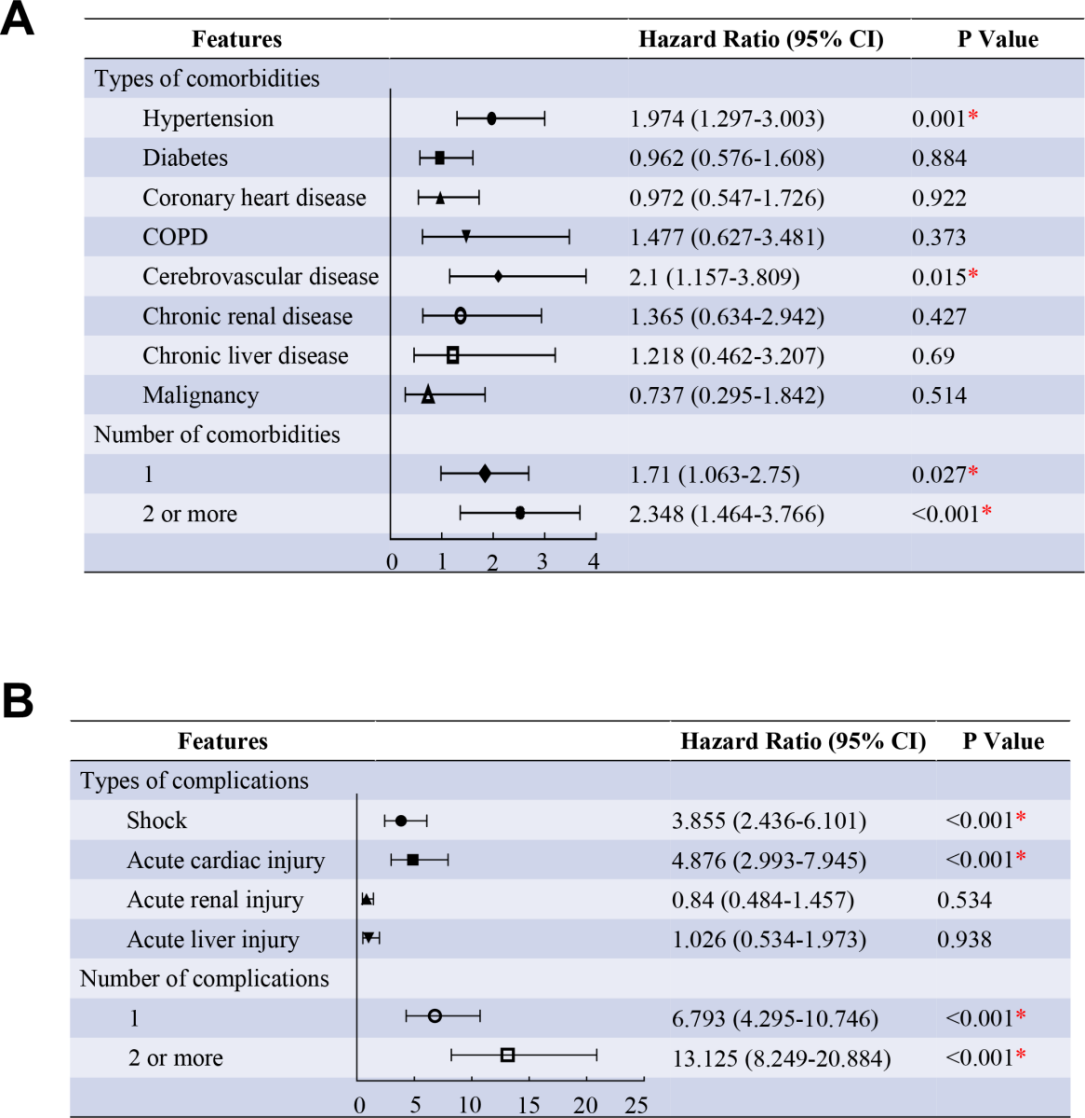
**Predictors of the death in the proportional hazards model.** (**A**) Shown in the figure are the hazards ratio (HR) and the 95% confidence interval (95%CI) for the risk factors of death after disease onset. The comorbidities were classified according to the organ systems as well as the number. (**B**), Shown in the figure are the hazards ratio (HR) and the 95% confidence interval (95%CI) for the risk factors of death after disease onset. The complications were classified according to the organ systems as well as the number. * means the P value <0.05. The scale bar indicates the HR. The model has been adjusted with age groups.

Age and comorbidities were incorporated into the proportional hazards model, and the analysis showed that patients with hypertension (HR, 1.974, 95% CI, 1.297-3.003), cerebrovascular disease (HR, 2.1, 95% CI, 1.157-3.809) were more likely to die than those without. As compared with patients without comorbidity, the HR (95% CI) was 1.71 (1.063-2.75) among patients with one comorbidity and 2.348 (1.464-3.766) among patients with two or more comorbidities after adjusting for age groups (Figure 2A). Another proportional hazards model incorporating age and complications showed that patients with acute cardiac injury (HR, 4.876, 95% CI, 2.993-7.945), shock (HR, 3.855, 95% CI, 2.436-6.101) were more likely to die than those without. As compared with patients without complications, the HR (95% CI) was 6.793 (4.295-10.746) among patients with only one complication and 13.125 (8.249-20.884) among patients with two or more complications after adjusting for age groups (Figure 2B). (all P <0.05)

## DISCUSSION

In this study, we found that age had a significant impact on the clinical characteristics and outcomes of COVID-19 patients. The symptoms of the elderly patients were more atypical than those of the young patients and were characterized by more comorbidities. More older patients had organ damage, immune dysfunction, and more severe inflammation on admission. During hospitalization, more older patients received oxygen therapy and experienced more complications. Most importantly, older patients were more likely to develop critical illness with a significantly higher mortality rate. The previous literature [10] discussed the mortality rate of COVID-19 patients at different ages by constructing models. It was speculated that the disease in Wuhan was more serious and that the mortality rate in the elderly population was higher. Our research further supports these views, and we found that the case fatality rate was significantly higher than that reported in this literature, which may be related to the fact that we did not include other patients, such as patients in mobile cabin hospitals. However, we described the clinical characteristics of patients at different ages and analyzed the relevant mechanisms that led to poor prognosis, which are complementary to the findings of the previous study [10].

According to the results of the study, we found that older patients, especially super-aged patients, may have atypical symptoms, and it may be harder to accurately identify older patients with COVID-19. Similar to previous reports [7, 15], the most common symptom was fever. However, the rate of fever in super-aged patients was significantly lower than that in younger patients. More elderly people showed symptoms of expectoration, dyspnea and chest tightness, which may be considered symptoms of underlying diseases such as COPD or coronary heart disease. The proportion of older patients with muscle pain decreased as they aged. Therefore, the symptoms of older patients seem to be more atypical than those of younger patients, making it harder to identify SARS-CoV-2 infection early. The results showed that the older patients presented with a slightly longer duration from the onset of symptoms to admission. A previous study showed that the main CT findings were ground-glass opacities and nodules [16]. However, our study found that super-aged patients showed a lower proportion of ground-glass opacities but more paving stone/reticular/linear findings than younger patients. It was suggested that CT images of super-aged patients may not be as typical as those of younger patients, and CT diagnosis alone was prone to misdiagnosis. It should be noted that the elderly with atypical symptoms may be missed, leading to the spread of infection. Therefore, the diagnosis of the elderly should be more careful.

Deaths and serious consequences in older patients have also been reported for other human coronaviruses, such as HCoV-OC43 [17], SARS-CoV [3], and MERS-CoV [18]. Older age has been reported as an important independent risk factor for mortality in SARS [19] and MERS [20]. A recent study confirmed that increased age was associated with death in patients with COVID-19 [14]. However, the mechanisms between poor prognosis and older age remain unclear.

After comparing the characteristics of COVID-19 patients of different ages, we found that the poor prognosis may be related to the higher proportion of comorbidities in older patients. In this study, the major comorbidities were hypertension, diabetes, and CHD. The proportion of old patients with comorbidities was much higher than that of young patients. The super-aged patients were more likely to have more than one comorbidity. Old patients with chronic comorbidities were more sensitive to SARS-CoV-2 because metabolic diseases were reported to lead to weaker immune functions [21]. A series of studies showed that the combined comorbidities were one of the independent risk factors of the poor prognosis of COVID-19 patients [14, 22–24]. Besides, our study reported that the comorbidities were an independent risk factor of death. The poor outcome of the older COVID-19 patients may be associated with the higher rate of comorbidities.

In addition, comorbidities such as hypertension, CHD and COPD lead to cardiac and pulmonary dysfunction. The deteriorated lung and cardiac function in the elderly may be associated with poor prognosis. With increasing age, changes in the anatomy of the lungs and muscle atrophy in the elderly lead to changes in the physiological functions of the respiratory system, reduced airway clearance, reduced lung reserve, and reduced barrier function. As shown in the results, the proportion of patients with dyspnea in the older age group was significantly higher than that in the young age group. The arterial oxygen saturation and arterial partial pressure of oxygen decreased with age. The proportion of patients receiving oxygen therapy regardless of nasal catheter, mask, or noninvasive mechanical ventilation in the older group was significantly higher than that in the younger group. These results indicate that older patients were more likely to develop worse conditions. Although we did not determine the number of patients who died of respiratory failure or heart failure, there was a report indicating that heart failure was observed in addition to respiratory failure in the patients who died [23].

In addition, we found that the proportion of patients with complications increased with age. Older patients are more likely to have more than one complication, especially super-aged patients. Complications such as cardiac injury were reported to be related to the poor prognosis of COVID-19 patients [12, 25–27]. And our result showed that the complications were an independent predictor of fatality. Therefore, the high mortality of older patients may be associated with a higher rate of complications.

This study showed that increases in nonspecific inflammation biomarkers, including CRP, hs-CRP, serum amyloid protein and procalcitonin, were more likely to occur in older patients. The proportion of patients with increased white blood cells and neutrophils in the older age groups was significantly higher than that in the younger age group. These findings suggest that older patients may be more likely to have a secondary infection with other bacteria, which may lead to poor prognosis [28]. Kim et al. reported that viral-bacterial coinfection was an independent predictor of mortality from viral pneumonia [29]. Although there was no difference in antibiotic treatment between the age groups, we believe that older patients need antibiotics more to prevent coinfection. Coinfection may be a risk factor for poor prognosis in older patients [30], but more research is needed to confirm this hypothesis.

Moreover, we speculate that the poor prognosis of older patients was related to the aging of the immune system. Immune system aging is an important process in the human aging process and is mainly manifested by the progressive degeneration of innate and adaptive immunity [31]. Because of immune aging, older patients are more susceptible to and impacted by bacteria and viruses, especially with comorbidities such as COPD. Previous research on SARS-CoV-inoculated macaques has found that older macaques had a stronger innate response to virus-infected hosts and increased expression of genes related to inflammation [32]. Defects in age-dependent T and B cell function and overproduction of type 2 cytokines may lead to inadequate viral replication control and longer pro-inflammatory responses, which may lead to adverse results [33]. In this study, we observed that the rate of lymphopenia was higher in the older age groups than in the younger age group. The function of humoral and cellular immunity was significantly downregulated in older patients. The proportion of patients receiving immunoglobulin therapy increased significantly with age. The expression level of IL-6 in super-aged patients was significantly increased. These results suggest that the immune system and inflammatory reaction of COVID-19 patients were disturbed. It was reported that SARS-CoV-2 may mainly affect T lymphocytes, especially CD4+ T cells, resulting in a significant decrease in lymphocyte numbers [34]. The extent of lymphopenia and increase in inflammatory cytokines were related to the severity of the disease [35]. Therefore, we speculate that the poor prognosis of the older patients was associated with the disturbed immune system and inflammation.

In summary, the high case fatality rate of elderly patients was related to comorbidities, reduced heart and lung function, complications, secondary infections and disturbed immune system and inflammation.

There are several limitations to our study. First, the follow-up period was short, and data on the outcomes of many patients were not collected. An extended follow-up period may help us better understand the prognosis of older patients. Second, this study is a retrospective study. We analyzed only the initial laboratory results. The dynamic changes in different markers during hospitalization should be further analyzed. Third, this study is only a descriptive study, and the mechanisms underlying the relationship between poor prognosis and age require further research. Fourth, this study is a single-center study. Patients included in the study were all from Renmin Hospital. And they were mainly severe and critically ill patients.

## MATERIALS AND METHODS

### Study design and participants

This is a retrospective study and was approved by the Ethics Commission of Renmin Hospital of Wuhan University. Data from a total of 1000 confirmed COVID-19 patients admitted to both the Shouyi and East districts of Renmin Hospital of Wuhan University from January 1, 2020 to February 14, 2020 were collected. The patients’ age ranges from 21 to 101 years; According to age, patients were divided into three groups: age group 1 (<60 years old), age group 2 (60-74 years old) and age group 3 (≥75 years old).

### Diagnostic criteria

The diagnosis of COVID-19 were performed according to The Diagnosis and Treatment Guidelines of Pneumonia Caused by Novel Coronavirus (6^th^ trial edition) published by the General Office of the National Health Commission and the General Office of the National Administration of Traditional Chinese Medicine [36].

Confirmed cases should be suspected cases with one of the following etiological evidences: 1) positive nucleic acid test; 2) sequencing of viral genes, highly homologous to known SARS-CoV-2.

The diagnosis of suspected cases needs to be combined with the following comprehensive analysis of epidemiological history and clinical manifestations. Epidemiology history: 1) travel history or residence history of Wuhan city and surrounding areas, or other communities with case reports within 14 days before onset; 2) had a history of contact with SARS-CoV-2 infection (positive nucleic acid test) within 14 days before onset; 3) had a history of contact with patients with fever or respiratory symptoms from Wuhan and surrounding areas, or from communities with case reports within 14 days before onset; 4) clustering onset of COVID-19 infection. Clinical manifestations: 1) fever or respiratory symptoms; 2) with the imaging features of COVID-19; 3) with normal or decreased number of white blood cells and reduced lymphocyte count in the early stage. Patients who met one of the following conditions were defined as suspected cases: 1) meet any one in the history of epidemiology and any two in the clinical manifestations; 2) no definite epidemiological history, but with all the three items in the clinical manifestations.

### Evaluation of clinical results

The onset of a disease was defined as the time when the associated symptoms first appeared. The outcome information of these patients was collected until February 24, 2020, including whether they were still in the hospital, improved and were discharge or were transferred to the isolation area for continued isolation and death.

According to the sixth edition of the Novel Coronavirus Pneumonia Diagnosis and Treatment Plan, the disease is generally classified into four types: mild, moderate, severe, and critical. Patients who met one of the following conditions were defined as severe: 1) dyspnea, breathing frequency >30 times per minute or 2) finger oxygen saturation ≤ 93%. Patients who met any of the following conditions were defined as critical: 1) respiratory failure requiring mechanical ventilation; 2) vibration; and 3) any concomitant organ failure other than respiratory failure, requiring monitoring and treatment in the intensive care unit (ICU). Other patients were classified as mild-moderate.

### Criteria for target organ injury

Plasma hypersensitivity troponin I (hs-TnI) levels above the 99% reference line were considered to indicate acute heart injury. Alanine aminotransferase (ALT≥150 U/L) increasing to three-fold higher than normal was considered to indicate acute liver injury (ALI). Patients with one of the following conditions could be diagnosed with acute kidney injury (AKI): 1) the highest serum creatinine (Scr) level increased by more than 26.5 μmol/L (0.3 mg/dL) within 48 hours; 2) Scr exceeded the baseline value by 1.5-fold (confirmed or estimated to occur within 7 days); and 3) urine output <0.5 ml/kg * h), lasting more than 6 hours. Patients with septic shock were clinically identified by vasopressor requirement to maintain a mean arterial pressure of 65 mmHg or greater and a serum lactate level greater than 2 mmol/L (>18 mg/dL) in the absence of hypovolemia [37].

### Data collection

Information including the patient's hospitalization, medical history, clinical symptoms, signs, laboratory tests, chest computed tomography (CT) scan, treatment, and outcome or prognosis was obtained through the hospital's medical record system. We analyzed the image of the first CT scan in our hospital within three days after admission. The first results of laboratory tests after admission were analyzed.

### Statistical analysis

Continuous variables are represented by medians and interquartile ranges (IQRs), and categorical variables are represented by numbers (percentages). The Mann-Whitney U test was used to analyze continuous variables. The χ^2^ test or Fisher’s exact test was used to analyze the classified variables. The Kaplan-Meier test was used to investigate the cumulative death rate among the three groups. Cox proportional hazard regression models were applied to determine the potential risk factors associated with the mortality, with the hazards ratio (HR) and 95% confidence interval (95%CI) being reported. The statistical software package of Social Sciences (SPSS 26.0) was used for analysis, and a P value < 0.05 was considered statistically significant.
